# A Matched Case‐Control Study to Evaluate Predicted Drug Exposures and Neutropenia during Valganciclovir Prophylaxis in Pediatric Solid Organ Transplant Recipients

**DOI:** 10.1111/tid.70146

**Published:** 2025-12-09

**Authors:** Mai‐Uyen T. Nguyen, Michael N. Neely, Anders Åsberg, Craig L. K. Boge, Kevin J. Downes

**Affiliations:** ^1^ Division of Infectious Diseases Children's Hospital of Philadelphia Philadelphia Pennsylvania USA; ^2^ Department of Pharmacology Physiology & Cancer Biology Thomas Jefferson University Philadelphia Pennsylvania USA; ^3^ Division of Infectious Diseases Children's Hospital of Los Angeles Los Angeles California USA; ^4^ Department of Pediatrics Keck School of Medicine, University of Southern California Los Angeles California USA; ^5^ Department of Pharmaceutical Biosciences School of Pharmacy University of Oslo Oslo Norway; ^6^ Department of Transplantation Medicine Oslo University Hospital, Rikshospitalet Oslo Norway; ^7^ Department of Pediatrics Perelman School of Medicine, University of Pennsylvania Philadelphia Pennsylvania USA

**Keywords:** cytomegalovirus, drug toxicity, ganciclovir, outcomes, pediatric transplant, pharmacokinetics

## Abstract

**Background:**

Neutropenia during valganciclovir (VGCV) prophylaxis for cytomegalovirus infection in pediatric solid organ transplant (pSOT) recipients is common, but it is uncertain if this toxicity is exposure‐dependent.

**Methods:**

To compare ganciclovir (GCV) exposures in children treated with VGCV with and without neutropenia, we performed a retrospective matched case‐control study among pSOT prescribed VGCV, dosed based on body surface area. Cases were defined as an absolute neutrophil count (ANC) < 1000/µL. Controls without neutropenia were matched by age (+/−1 year), transplanted organ, and duration of VGCV prophylaxis. We used a published population pharmacokinetic model to inform predictions of GCV concentrations using Pmetrics, accounting for each subject's time‐dependent variables (age, weight, creatinine clearance). We then calculated 24‐h, 7‐day, and cumulative area under the curve (AUC) in each subject and used conditional logistic regression to compare GCV exposures among cases and controls.

**Results:**

Among 164 pSOT recipients prescribed VGCV, we identified 35 case‐control matches. There were no statistically significant differences in the 24‐h (odds ratio [OR] 0.990, 95% confidence interval [CI] 0.964–1.018), 7‐day (OR 1.000, 95% CI 0.996–1.004), or cumulative AUCs (OR 1.00, 95% CI 0.9996–1.00) among all cases and controls. AUC metrics by SOT type also showed no statistically significant differences.

**Conclusions:**

Predicted GCV exposures were similar among pSOT recipients with and without neutropenia, suggesting that differences in dosing and pharmacokinetics covariates did not drive toxicity in our population. Measurement of GCV concentrations may discern whether toxicity relates to exposures/concentrations or intrinsic factors (i.e., genetics) in the pSOT population.

## Graphical abstract

In this retrospective case‐control study, among 35 case‐control matches, no differences were identified in predicted plasma ganciclovir AUCs between pediatric solid organ transplant (SOT) recipients prescribed valganciclovir with and without neutropenia. Interindividual variability in pharmacokinetic or pharmacodynamic factors (e.g., genetic susceptibility) among pediatric SOT recipients may influence neutropenia development.

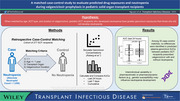

**Abbreviations**
ANC: absolute neutrophil count
ATG: anti‐thymocyte globulin
AUC: area under the curve
BSA: body surface area
CHOP: Children's Hospital of Philadelphia
CI: confidence interval
Cmax: maximal concentration
CMV: cytomegalovirus
GCV: ganciclovir
GFR: glomerular filtration rate
OR: odds ratio
PK: pharmacokinetic
pSOT: pediatric solid organ transplant
SOT: solid organ transplant
TDM: therapeutic drug monitoring
VGCV: valganciclovir

## 1 Introduction

Solid organ transplant (SOT) recipients are at high risk of developing infectious complications. Cytomegalovirus (CMV) is the most common opportunistic pathogen, but routine antiviral prophylaxis effectively reduces symptomatic disease. Ganciclovir (GCV) and its oral prodrug formulation, valganciclovir (VGCV), are antiviral drugs frequently administered for CMV prevention and treatment. While effective use of GCV and VGCV is associated with high rates of toxicities, especially neutropenia, in pediatric solid organ transplant (pSOT) patients [1, 2]. Although neutropenia in pSOT is often multifactorial, prophylaxis with VGCV is associated with a four times higher rate of neutropenia compared to no antiviral prophylaxis [1]. Furthermore, the prevalence of neutropenia during VGCV prophylaxis after transplant varies across institutions, with approximately 30% at best [3, 4], but ranging up to nearly 70% [5], which may be related to VGCV dosing strategies.

VGCV dosing in pSOT recipients is typically based on either body weight or body surface area (BSA). Controversies exist as to the preferred approach, as BSA‐based dosing can lead to more frequent dose‐dependent toxicities, but body weight‐based dosing may result in lower exposures that could increase the risk of treatment failure [4, 6, 7, 8]. VGCV dosing approaches are further complicated by the lack of routine therapeutic drug monitoring (TDM). In a pharmacokinetic (PK) study that dosed VGCV based on body weight and glomerular filtration rate (GFR) using the modified Schwartz equation, plasma concentration area under the curve (AUC) exposures were outside of the therapeutic target range of 40–60 µg · h/mL in 39% of pSOT recipients [8]. These issues collectively call attention to variable VGCV exposures across pSOT recipients for CMV prophylaxis, and such variability could contribute to neutropenia development.

We previously conducted a retrospective cohort study at the Children's Hospital of Philadelphia (CHOP) that evaluated the incidence of VGCV‐associated toxicities, including kidney injury, leukopenia, and neutropenia, in pSOT recipients [1]. For the current study, we used this cohort to determine whether VGCV dosing or plasma concentrations predicted from patient characteristics were related to neutropenia. We hypothesized that, when matched by age, SOT type, and duration of VGCV, pSOT recipients who developed neutropenia would be expected to have greater GCV exposures than those who did not develop neutropenia. To evaluate this hypothesis, we used a previously published population model [9] to generate predicted GCV exposures among recipients of VGCV and compared exposures among those with and without neutropenia using a retrospective, matched case‐control design.

## 2 Methods

### 2.1 Study Population

A cohort of pSOT recipients at CHOP who were identified for a previous study [10] served as the basis for the current analysis. All patients received BSA‐based dosing, capped at a modified Schwartz estimated GFR of 120 mL/min/1.73 m^2^, as was institutional protocol. Those who received a first heart, lung, liver, or kidney transplant between January 2012 and June 2018 and were less than 19 years of age at SOT were eligible for inclusion if they received prophylactic doses of VGCV after SOT, defined as once daily dosing of VGCV in the absence of CMV DNAemia. Patients who developed neutropenia while on treatment with VGCV (twice daily dosing) for CMV infection were excluded. Because neutropenia in the first 2 weeks post‐transplant was likely multifactorial rather than directly attributable to drug toxicity, only individuals who received VGCV for more than or equal to 2 weeks were considered for the study. This study was approved by the CHOP IRB (#23‐021644).

### 2.2 Cases, Controls, and Matching

Cases were defined as individuals who developed neutropenia (at least one absolute neutrophil count [ANC] measurement of < 1000/µL) while taking VGCV prophylaxis. Cases were censored on the day of neutropenia toxicity and must have had at least one preceding ANC value of at least 1500/µL. All individuals who did not develop neutropenia at any time during VGCV prophylaxis were eligible to serve as controls. Cases and controls were matched 1:1 without replacement by SOT organ type, age at the start of VGCV prophylaxis (+/−1 year), and VGCV duration in days. Matched controls could have received VGCV for a longer duration than cases, but control courses were censored so that case‐control pairs had the same duration (in days) of prophylaxis. Matching was conducted with the MatchIt library package (version 4.5.5) in R using the default method (nearest neighbor matching) for VGCV exposure duration, exact matching for SOT type, and caliper matching (based on raw units instead of standard deviation) for age.

To determine the duration of VGCV exposure, the number of consecutive days that an individual took VGCV was tallied. Gaps in VGCV therapy of more than or equal to 7 days were considered new courses of prophylaxis, and only the block of prophylaxis that immediately preceded neutropenia onset (for cases) or the longest block of prophylaxis (for controls) was included. Gaps in VGCV administration of less than seven consecutive days were included in the total time of VGCV exposure under the assumption that VGCV was not completely washed out intracellularly within that time frame. The 7‐day gap was selected in consideration of both the elimination half‐life of oral VGCV in pSOT recipients, ranging approximately 3–6 h, on average [11], and the kinetics of white blood cell recovery following VGCV cessation [12].

### 2.3 Data Management

Patient demographic, laboratory, and medication data were acquired for this study from CHOP's electronic medical record, including age, sex, height, weight, serum creatinine, ANC, and VGCV dosing data from transplant through 1‐year post‐transplant. We generated a dataset with each row corresponding to the days of VGCV administration that contains information for VGCV dosage, age, weight, and sex for each case and control. For each subject, any missing measurements for weight, height, and serum creatinine on a given day were filled by carrying forward the last recorded value available (i.e., if weight was measured weekly, that weight was applied for 7 days until the next measurement was performed). If no measurement was available at the start of VGCV, then the first recorded value was applied (i.e., carrying backward). Plasma VGCV concentrations were not available and not required for this study. Data cleaning and formatting was completed in R (version 2024.04.2) using the library packages dplyr (version 1.1.4) and tidyr (version 1.3.1).

Concomitant medications were categorized based on use relative to the day of neutropenia onset or day of censoring for each matched case and control, respectively, as a reference point. Each daily immunosuppressive medication and trimethoprim‐sulfamethoxazole was dichotomized (yes/no) based on whether the case/control had an active prescription for the medication on the day of neutropenia onset, censoring (see above for censoring definitions), as well as within 7 days of neutropenia onset or censoring. Longer‐acting conditioning agents (alemtuzumab, anti‐thymocyte globulin [ATG], and basiliximab) were classified based on administration within 180 days of neutropenia onset or censoring.

### 2.4 Simulating GCV Concentrations

A population PK model for VGCV in pSOT recipients was previously developed by Åsberg et al. [9]. This model was created using the nonparametric modeling software package for R, Pmetrics (version 2.1.1, Laboratory of Applied Pharmacokinetics, Los Angeles, California) [13]. The final model was a two‐compartment model with bioavailability and lag time parameters. The model incorporated creatinine clearance, estimated with the Cockcroft–Gault equation for all ages [14], as a covariate on GCV/VGCV clearance and allometric scaling on all parameters.

The final parameter value probability distributions were used to simulate (predict) GCV concentrations for cases and controls in the current study. To prepare data for simulations, we modified the daily dataset to contain information for simulations (VGCV dosage, age, weight, creatinine clearance, and sex) for each case and control. The dataset was then limited to one row (or record) every 7 days for each patient, to both facilitate longitudinal simulations and balance the frequency of changes in time‐varying measurements (age and creatinine clearance) across individuals. Our study also calculated creatinine clearance using the Cockcroft–Gault equation to maintain consistency with the Åsberg model.

To estimate GCV concentrations for cases and controls, Monte Carlo simulations were performed with Pmetrics in R. We used the weighted medians of the parameter values from the Åsberg model, along with each subject's dosing and covariate data, to simulate one concentration‐time profile per subject. Due to the limited capacity on the number of simulated observations (i.e., concentrations) in Pmetrics, simulations were broken down into smaller blocks. Specifically, each run simulated GCV concentrations every hour in 504‐h blocks, as needed, until the full GCV plasma concentration‐time profile was generated based on the duration of each case‐control pair. Cumulative AUC over the full course of VGCV prophylaxis (cumulative AUC), AUC over the last 7 days before neutropenia onset or time of censoring for controls (7‐day AUC), and AUC from the last 24 h before neutropenia onset (24‐h AUC) were calculated for each subject based on the simulated concentrations. Maximal concentration (C_max_) in the last 24 h of therapy prior to neutropenia/censoring was also derived from each subject's predicted concentrations.

### 2.5 Data Analysis and Visualization

Characteristics at the start of VGCV therapy and concomitant medications were summarized using standard descriptive statistics. We compared these characteristics, as well as use of concomitant medications, between cases and controls using chi‐square tests, Fisher's exact tests, or Wilcoxon rank‐sum tests, as indicated.

To describe exposures among cases and controls, we summarized the exposures in several ways. First, we evaluated the proportion of cases and controls that had a predicted (simulated) 24‐h AUC within, above, and below the goal range of 40–60 mg · h/L, which is the standard therapeutic target for VGCV/GCV during prophylaxis [15, 16]. Next, we calculated ratios of the 24‐h AUCs for each case‐control match (predicted AUC of the case divided by that of their respective matched control). We also summarized each exposure metric (AUC, C_max_) using medians and ranges. To visually display these data, graphs were generated with the library package ggplot2 (version 3.5.1) in R. No statistical tests were performed when exposures were summarized as described above.

Finally, we compared exposures among cases and controls using conditional logistic regression. Separate analyses were conducted for each of the AUC exposure metrics: 24‐h AUC, 7‐day AUC, and cumulative AUC. Additional subset analyses were also performed within each of the organ groups (not including lung due to the inclusion of a single matched pair). For all conditional logistic regression tests, the clogit function was used from the R library package survival (version 3.6.4).

## 3 Results

### 3.1 Study Population

**FIGURE 1:**
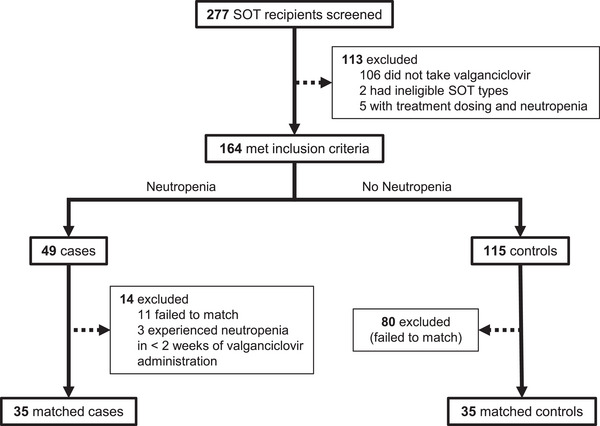
Flow chart for subject eligibility and matching. Neutropenia is defined as an ANC measurement of < 1000/µL after at least 2 weeks of valganciclovir prophylaxis, but also has any preceding ANC value of at least 1500/µL.

From the cohort of 277 pediatric SOT recipients at CHOP, 106 did not receive VGCV, 2 subjects received a SOT type that was not eligible for the study, and 5 developed neutropenia with treatment dosing for VGCV (Figure 1). Of the remaining 164 children, 49 developed neutropenia (30%) during VGCV prophylaxis and were potential cases. Three cases were dropped due to neutropenia onset within 2 weeks of transplant, and 11 cases did not have a suitable match. Ultimately, we successfully matched 35 cases to controls (14 kidney, 12 liver, 8 heart, and 1 lung SOTs) based on our matching criteria. The median time to toxicity for cases was 74 days (range 19–273). A total of 14 cases (40%) and 13 controls (37.1%) were female (chi‐square test, *p *= 1.00). The 11 cases of neutropenia who did not have an available match were older (median age of 10 years) and had a later onset of neutropenia (median 99 days) compared to matched cases; three of four lung transplant recipients with neutropenia did not have a suitable age‐ and VGCV duration‐matched control due to a limited pool of lung transplant recipients at our institution.

Characteristics at the start of VGCV therapy, including weight, BSA, creatinine clearance, estimated GFR, initial dosing, and sex, were similar between cases and controls (Table 1). All cases and matched controls took at least one immunosuppressive drug on the day of neutropenia or censoring (Table 1) as well as within the last 7 days (data not shown). There were no statistically significant differences in the conditioning agents administered within 180 days prior to neutropenia onset or censoring. Both matched cases and controls took a median of two immunosuppressive drugs (range 1–3, Wilcoxon rank‐sum test, *p* = 0.96). Overall, no statistically significant difference was observed for any concomitant medication at any time point that was assessed.

**TABLE 1:** Characteristics of matched subjects.

Characteristic	Case	Control	*p *value
**At start of valganciclovir therapy**			
Age (years)^a^	5.4 (0.6–18.8)	4.6 (0.1–18.3)	0.83
Weight (kg)^a^	18.6 (5.9–74.4)	16.9 (4.2–66.2)	0.91
Body surface area (m^2^)^a^	0.73 (0.30–1.94)	0.70 (0.25–1.76)	0.90
Cockcroft–Gault CrCl (mL/min)^a^	71 (17–189)	84 (7–202)	0.53
Modified Schwartz eGFR (mL/min/1.73 m^2^)^a^	129 (17–305)	146 (9–312)	0.42
Daily dose^a^			
mg/dose	500 (112–900)	550 (100–900)	0.62
mg/kg/dose	29.3 (1.5–50.0)	33.1 (2.6–52.3)	0.42
mg/kg/m^2^/dose	42.8 (0.8–148.9)	51.8 (1.8–178.1)	0.72
Receipt of maximal VGCV dose of 900 mg/dose (*n* [%])^b^	9 (25.7)	12 (34.3)	0.60
Days between SOT and VGCV initiation^a^	6 (2–71)	7 (2–72)	0.45
Time to toxicity/censoring after transplant (days)^a^	88 (25–276)	84 (39–280)	0.94
**Concomitant medications (*n* [%])**			
**On day of neutropenia/censoring**			
Immunosuppressive and anti‐infective prophylaxis agents			
Azathioprine^c^	3 (8.6)	0 (0)	0.24
Mycophenolate mofetil^b^	23 (65.7)	25 (71.4)	0.80
Sirolimus^c^	1 (2.9)	3 (8.6)	0.61
Tacrolimus^c^	35 (100)	34 (97.1)	1.00
Trimethoprim‐sulfamethoxazole^b^	25 (71.4)	27 (77.1)	0.78
**Within 180 days of neutropenia/censoring**			
Conditioning agents			
Anti‐thymocyte globulin^b^	22 (62.9)	22 (62.9)	1.00
Basiliximab^c^	1 (2.9)	1 (2.9)	1.00

*Note*: Median (range) shown for all values unless indicated otherwise. Creatinine clearance was calculated with the Cockcroft–Gault equation. Body surface area was calculated with the Mosteller formula.

Abbreviations: CrCl, creatinine clearance; eGFR, estimated glomerular filtration rate; SOT, solid organ transplant; VGCV, valganciclovir.

^a^ *p* values were calculated using the Wilcoxon rank‐sum test.

^b^ *p* values were calculated using the chi‐square test.

^c^ *p* values were calculated using the Fisher's exact test.

### 3.2 VGCV Exposures and Neutropenia

The median predicted 24‐h AUC was 68.6 mg · h/L (range 15.1–96.6 mg · h/L) for cases and 65.2 mg · h/L (range 18.9–112.8 mg · h/L) for controls. For the 11 unmatched cases, the median predicted 24‐h AUC was similar to that of the matched cases (Wilcoxon *p* value = 0.51). Similar proportions of the cases and controls had predicted 24‐h AUCs within the range of 40–60 mg · h/L: 10 (28.6%) cases versus 12 (34.3%) controls. For both cases and controls, 19 (54.3%) had predicted AUCs more than 60 mg · h/L, while 6 cases (17.1%) and 4 controls (11.4%) were less than 40 mg · h/L. Predicted C_max_ in the last 24 h prior to neutropenia onset (or censoring for controls) was also similar among cases (median 12.1 mg/L, range 2.5–17.0 mg/L) and controls (median 12.5 mg/L, range 3.1–19.8 mg/L). The median 24‐h AUC ratio between cases and their respective control match was 0.91 (range 0.33–3.13).

**FIGURE 2:**
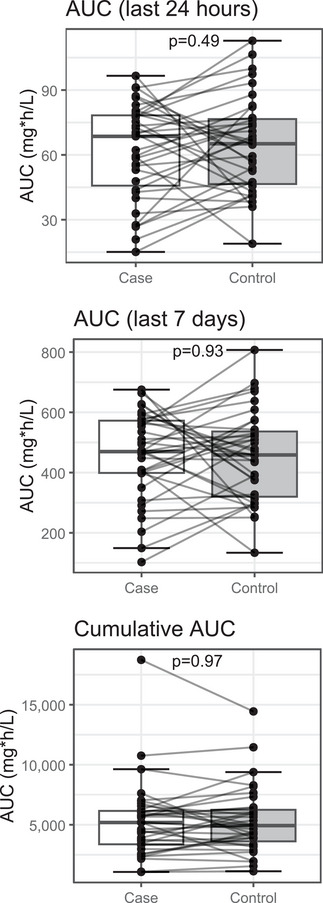
Predicted ganciclovir AUCs do not differ between cases and controls. AUC of the case and control groups for the last 24 h (top), last 7 days (middle), and entire treatment duration (cumulative, bottom) before neutropenia. AUCs are derived from simulated GCV concentrations. Boxes indicate the 25th–75th percentile with the middle line as the median. Whiskers cover data points within 1.5 times the interquartile range from the lower and upper quartiles. Each point represents an individual subject, which is connected to their matched case or control with a gray line. *p *values were calculated with conditional logistic regression.

Based on conditional logistic regression analyses, there were no statistically significant differences in the expected 24‐h, 7‐day, or cumulative AUCs among cases and controls (Figure 2, Table 2). Similarly, we did not identify differences in any of the AUC metrics among cases and controls when analyses were performed separately for each organ group (Figure S1).

**TABLE 2:** Conditional logistic regression for AUC metrics. Odds ratios are shown with 95% confidence intervals for cases versus controls.

	OR (95% CI)	*p* value
Last 24 h	0.9904 (0.9635–1.0180)	0.49
Last 7 days	1.0002 (0.9962–1.0040)	0.93
Cumulative	1.0000 (0.9996–1.0000)	0.97

Abbreviations: CI, confidence interval; OR, odds ratio.

## 4 Discussion

Using a case‐control design, we did not identify differences in expected GCV AUCs obtained by simulation using individual subjects’ doses and relevant characteristics (model covariates) among pSOT recipients with and without neutropenia during VGCV prophylaxis. Expected GCV plasma concentration‐time profiles were similar after matching by age, SOT type, and duration of prophylaxis. This finding suggests that, among individuals administered a similar dosing strategy (i.e., all given BSA‐based dosing), VGCV‐induced neutropenia may not be an exposure‐driven toxicity. In our study population, covariates and characteristics at the start of VGCV therapy between matched cases and controls were similar and did not appear to influence the development of neutropenia. Alternatively, it is possible that exposures and neutropenia risk cannot be fully discriminated without the measurement of GCV concentrations.

A recent study in pSOT recipients by Gerthoffer and colleagues reported that VGCV dose, accounting for both BSA and kidney function, was associated with increased odds of neutropenia, but that VGCV dosing in milligrams per kilogram was not [3]. We did not appreciate differences in dosing by daily dose or weight‐adjusted daily dose in our study population. Kidney function and BSA were the primary drivers of dosing (and hence predicted GCV plasma profiles) in our study population, yet we did not identify differences in either BSA, kidney function (estimated with either Cockcroft–Gault or modified Schwartz equations), or the resulting AUCs across cases and controls. The Gerthoffer study estimated GFR with a cystatin C‐based formula [3], potentially leading to more individualized doses than were prescribed in our cohort based on BSA and serum creatinine‐based GFR. This highlights that, even across institutions using BSA‐based dosing, variability remains in dosing practices. Debate continues regarding which estimated GFR equation to follow and whether to employ a maximal cut‐off in the calculation to avoid exceedingly large doses.

VGCV demonstrates significant interindividual variability in PK among children [17, 18, 19]. Thus, concentrations often vary widely across individuals prescribed the same size‐adjusted dose. Importantly, we could not capture that variability in our study as we did not have measured GCV concentrations available to inform the actual AUCs that patients achieved. However, we hypothesized that variability in kidney function, body size, and dosages administered would contribute to differences in expected longitudinal GCV exposures among pSOT recipients with and without neutropenia. Although we did not find that to be true, our results are noteworthy and highlight that VGCV‐associated neutropenia is not predictable from these factors alone. Instead, there may be important drivers of toxicity that are only identifiable through the use of model‐informed precision dosing (i.e., measurement of GCV concentrations), recognition of alternative risk factors (e.g., use of other myelosuppressive medications), or genetic testing. For example, genetic polymorphisms in the *NUDT15* gene, which encodes an intracellular enzyme that helps break down certain thiopurine triphosphates like GCV may contribute to variability in intracellular GCV concentrations that may lead to neutropenia toxicity [20]. We did not find any statistically significant differences in immunosuppressive regimens or use of trimethoprim‐sulfamethoxazole between cases and controls, but it is possible that there were other unmeasured differences between cases and controls in our study. Interestingly, based on our simulations, a majority of both cases and controls exceeded the typical therapeutic AUC range. Therefore, there may also be protective factors that reduce susceptibility to toxicity when supratherapeutic exposures are achieved.

Furthermore, the population PK model by Åsberg et al. [9] had population predictions with an *R*‐squared of 0.784, slope 0.918, intercept 0.151, and very low prediction bias at 0.175 mg/L. The robustness of this model justified its use for predictions in our study. However, 20% of the variability in an individual remains unexplained based on population predictions alone. Therefore, it is possible that exposure differences were present in our population but not detected due to the absence of GCV concentrations to facilitate individual predictions. Additionally, this model was developed in only renal and liver transplant recipients, and its applicability to heart and lung transplant recipients (as were included in our study) has not been evaluated. However, we see a similar trend in AUC versus age when BSA‐based dosing is used, as was found in the Åsberg et al. study (Figure S2). Therefore, despite the lack of available GCV measurements in our study, the exposures (AUCs) mirror those previously described.

TDM for VGCV/GCV is frequently proposed as a means to better understand how exposure relates to efficacy and toxicity, especially where interindividual variability exists, and ultimately guide dosing strategies. In adult SOT populations, high interindividual variability in GCV concentrations is typically observed with standard VGCV dosing: patients more often fall below the therapeutic range and fewer above [21, 22]. However, existing literature points to poor correlations between VGCV/GCV exposure metrics (e.g., AUC, C_max_), clinical efficacy, and toxicity.[21, 23, 24] Given the lack of robust studies that support current AUC exposure targets, the utility of TDM in adults has been challenged.[16, 25, 26, 27]

Fewer studies have been performed examining VGCV/GCV TDM in children. As in adults, high interindividual variability and low AUCs are commonly reported in children given recommended dosages of VGCV/GCV [16, 28]. Therefore, TDM may be justified in pediatric populations. In two case studies of pediatric hematopoietic stem cell recipients experiencing CMV treatment failure, one of whom developed drug‐resistant CMV, TDM of GCV prompted dose adjustments that achieved therapeutic response and promoted drug efficacy [29, 30]. However, current literature employing TDM in children has not explored toxicity in relation to GCV concentrations. Meanwhile, model‐based simulations found an increasing probability of neutropenia with higher 24‐h AUC, but that risk only increased from 10% to 20% when moving from the AUC target range for prophylaxis to the treatment range (80–120 mg · h/L) [6]. In our study, subjects’ predicted 24‐h AUC often exceeded the putative target range for prophylaxis (40–60 mg · h/L), including in controls who did not experience toxicity. Without measured GCV concentrations available to us, however, we do not know where the true AUCs fell among patients in our study, and it is possible that differences existed between cases and controls that only would have been apparent had GCV been measured. Overall, TDM can be a tool to minimize variability across patients and define exposure‐response relationships. With routine use, TDM data can also potentially identify alternative TDM targets that better correspond to clinical outcomes for VGCV/GCV in pSOT [25, 27].

Our study has several limitations. First, as noted, we did not have measured GCV concentrations available to inform the actual AUCs achieved. Simulations were informed by the dosages prescribed, as well as serum creatinine, weight, and height measurements available. Additional studies are needed to understand how inter‐individual variability in GCV concentrations influences toxicity. Second, the majority of VGCV use in pSOT was in the outpatient setting. Thus, duration of VGCV administration was based on ordered dosing information. Patients may have missed more doses, which could affect toxicity risk, but that could not be captured in our retrospective design. Third, we used only one population PK model, though other models are available for estimating GCV in pSOT [31]. However, sex is the only additional covariate in these other models, which would not likely lead to any substantial differences compared to our findings. Last, because of our relatively small sample size and matching criteria, our findings may not be generalizable across all pSOT recipients. Older children receiving longer courses of VGCV prophylaxis, including lung transplant recipients, less often had available controls.

In conclusion, we did not identify differences in predicted plasma GCV AUCs between pSOT recipients prescribed VGCV with and without neutropenia. At our center, BSA‐based VGCV dosing, accounting for renal function, resulted in similar expected short‐ and long‐term GCV exposures among cases and controls. This finding suggests that other variability in interindividual PK or pharmacodynamic factors (e.g., genetic susceptibility) may influence neutropenia development in pSOT recipients. A larger study, in which GCV concentrations are available, is needed to tease out the relative contributions of dosing, exposures, and inherent susceptibility to toxicity in children.

## Funding

M.U.T.N. is supported by the NIH/NIGMS T32 Clinical Pharmacology Postdoctoral Training Program (T32GM008562).

## Conflicts of Interest

Kevin J. Downes receives research support from Paratek Pharmaceuticals, Inc. and Veloxis Pharmaceuticals, Inc., unrelated to this research. All other authors declare no conflicts of interest.

## Supporting information




**Supplementary Figure 1. Predicted ganciclovir AUCs among cases and controls categorized by solid organ transplant (SOT) type**. AUC of the case and control groups for the last 24 h (top), last 7 days (middle), and entire treatment duration (cumulative, bottom) before neutropenia categorized by each SOT. AUCs are derived from simulated GCV concentrations. Boxes indicate the 25th to 75th percentile with the middle line as the median. Whiskers cover data points within 1.5 times the interquartile range from the lower and upper quartiles. Each point represents an individual subject, which is connected to their matched case or control with a gray line. All p‐values > 0.05 for each SOT type and each timeframe (conditional logistic regression).
**Supplementary Figure 2. Predicted ganciclovir AUCs in the last 24 h versus age from two studies**. Left: Scatterplots of AUC vs age from the current study. Colored dots and lines represent predicted AUCs and the linear regression line for cases (pink) and controls (teal). Shaded regions indicate the 95% confidence interval for each linear regression line. Right: Notched boxplots of simulated AUC(0–24) values by age according to three different dosing algorithms: body surface area‐based dosing (blue), body weight‐based dosing (orange), and dosing algorithm defined by Åsberg et al (black). Figure reproduced with permission (license #6151361177481) from Åsberg et al. (2014), *Pediatric Transplantation*, Volume: 18, Issue: 1, Pages: 103‐111, DOI: (10.1111/petr.12179).

## Data Availability

Data for this study was generated as part of a Merck Investigator Studies Program (Downes, MISP‐57792).
