# Supplementary material for: A Matched Case‐Control Study to Evaluate Predicted Drug Exposures and Neutropenia during Valganciclovir Prophylaxis in Pediatric Solid Organ Transplant Recipients
Source: Transpl Infect Dis. 2025 Dec 9;28(1):e70146. doi: 10.1111/tid.70146 (PMC12892838; doi:10.1111/tid.70146)

SUPPLEMENTARY FIGURE 1

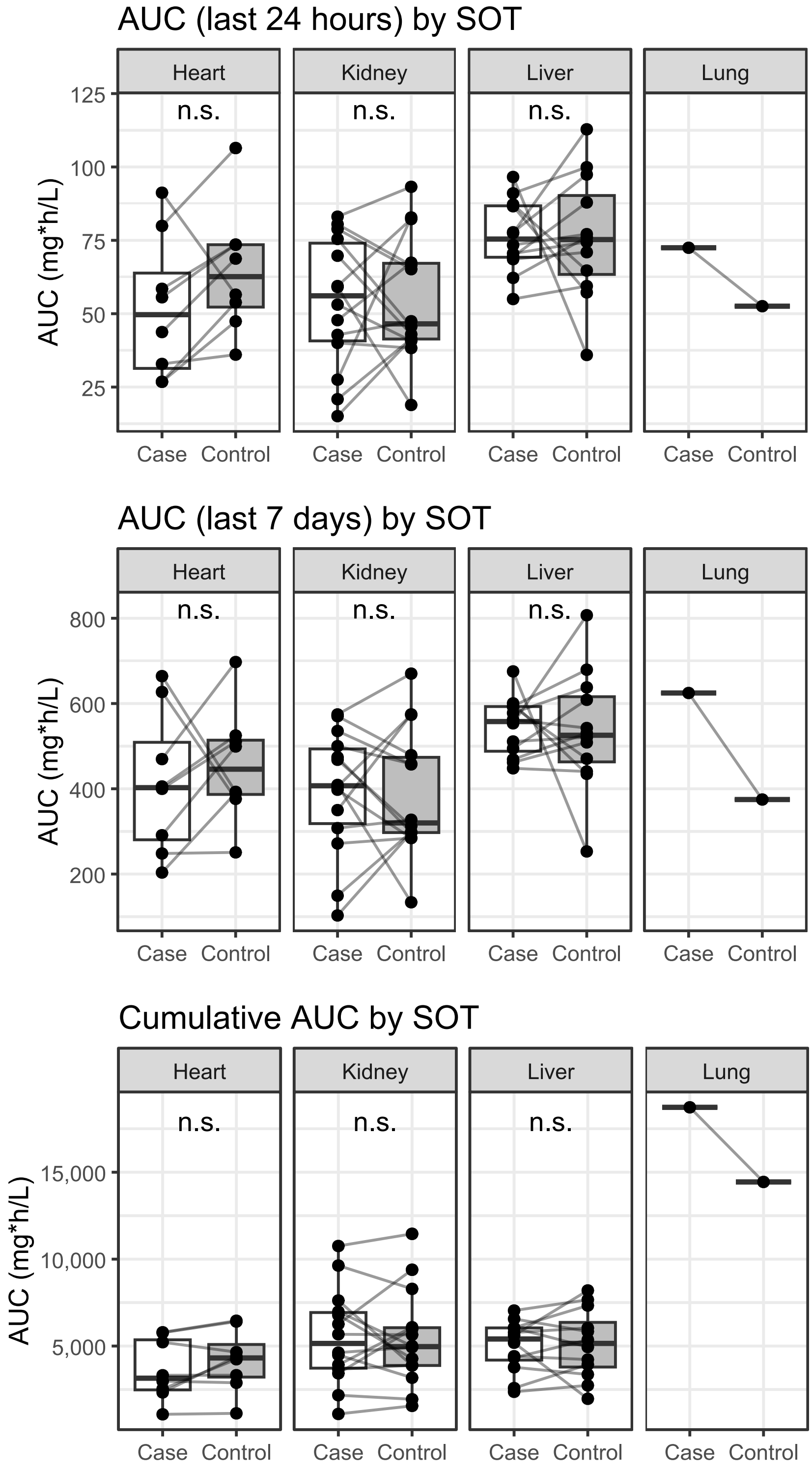

SUPPLEMENTARY FIGURE 2

Our Study

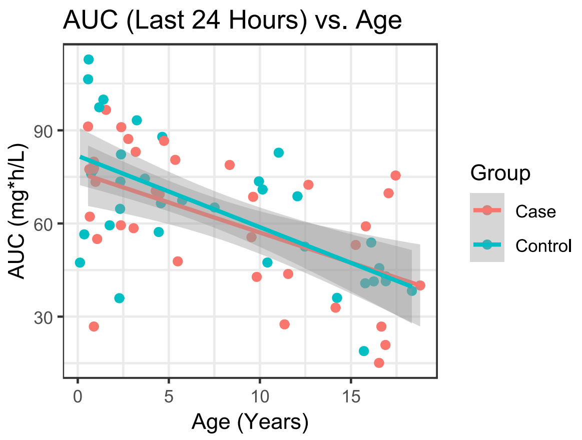

Åsberg et al. (2014)

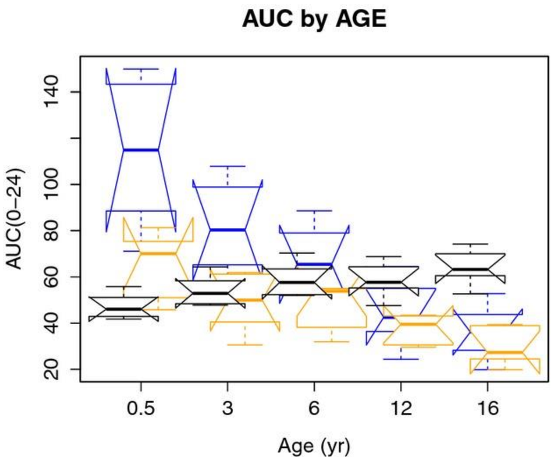

Supplement: Supplementary file 1 — Supplementary Figure 1. Predicted ganciclovir AUCs among cases and controls categorized by solid organ transplant (SOT) type. AUC of the case and control groups for the last 24 h (top), last 7 days (middle), and entire treatment duration (cumulative, bottom) before neutropenia categorized by each SOT. AUCs are derived from simulated GCV concentrations. Boxes indicate the 25th to 75th percentile with the middle line as the median. Whiskers cover data points within 1.5 times the interquartile range from the lower and upper quartiles. Each point represents an individual subject, which is connected to their matched case or control with a gray line. All p‐values > 0.05 for each SOT type and each timeframe (conditional logistic regression). Supplementary Figure 2. Predicted ganciclovir AUCs in the last 24 h versus age from two studies. Left: Scatterplots of AUC vs age from the current study. Colored dots and lines represent predicted AUCs and the linear regression line for cases (pink) and controls (teal). Shaded regions indicate the 95% confidence interval for each linear regression line. Right: Notched boxplots of simulated AUC(0–24) values by age according to three different dosing algorithms: body surface area‐based dosing (blue), body weight‐based dosing (orange), and dosing algorithm defined by Åsberg et al (black). Figure reproduced with permission (license #6151361177481) from Åsberg et al. (2014), Pediatric Transplantation, Volume: 18, Issue: 1, Pages: 103‐111, DOI: (10.1111/petr.12179). [file TID-28-e70146-s001.pdf]
